# Fractionated Stereotactic Radiation for Central Nervous System Lymphoma: Retrospective Analysis of Initial Cases

**DOI:** 10.3390/curroncol30090624

**Published:** 2023-09-20

**Authors:** Daniel G. Schep, Taskia Mir, Graeme A. M. Fraser, Jeffrey N. Greenspoon

**Affiliations:** Juravinski Cancer Centre, Hamilton, ON L8V 5C2, Canada; daniel.schep@medportal.ca (D.G.S.); taskia.mir@saskcancer.ca (T.M.); fraserg@hhsc.ca (G.A.M.F.)

**Keywords:** stereotactic radiation, radiosurgery, CNS lymphoma

## Abstract

Primary central nervous system lymphoma (PCNSL) is primarily treated with combination chemotherapy, while whole-brain radiotherapy (WBRT) can be used as consolidative treatment or as a salvage option for central nervous system (CNS) relapse. We investigated whether fractionated stereotactic radiosurgery (fSRS) could replace WBRT in cases where patients had poor performance status or minimal disease at the time of consolidation, to spare patients the adverse effects of WBRT. We retrospectively identified 10 patients who completed 14 courses of fSRS for PCNSL or for CNS relapse of systemic lymphoma. Of 14 fSRS treatments, there were 10 distant brain recurrences among 6 patients, occurring on average 13.6 months after fSRS. A total of 4 of the 10 recurrences were treated with further fSRS, and 4 were treated with WBRT. There was one late in-field recurrence after both fSRS and WBRT, which occurred 27 months after fSRS. The median survival after fSRS was 36 months, and side effects after fSRS were minimal. This case series represents a potential treatment option for patients with CNS lymphoma, for whom WBRT is indicated but where the toxic effects of this treatment would be prohibitive.

## 1. Introduction

Primary central nervous system lymphoma (PCNSL) is a rare malignancy that is treated primarily with combination chemotherapy [1]. While whole-brain radiotherapy (WBRT) has historically been used as a primary treatment for PCNSL, contemporary WBRT is an option for consolidation after induction chemotherapy [2]. Unfortunately, WBRT is associated with significant neurotoxicity, especially in patients over 60 years of age who are treated with chemotherapy regimens for PCNSL [3,4]. As a result of this toxicity, recent trials have examined omitting WBRT [5], reducing the dose of WBRT [6,7], or replacing consolidative WBRT with autologous stem-cell transplant (ASCT) [7,8]. Despite these investigations, WBRT is still a standard treatment for patients with PCNSL who have residual disease after initial systemic therapy, or who have CNS relapse of their disease [9,10].

Stereotactic radiosurgery (SRS) is a radiation technique that targets a specific region of the brain while sparing surrounding healthy parenchyma. When used to treat brain metastases, SRS results in less long-term neurotoxicity compared with WBRT [11]. Fractionated SRS (fSRS) indicates that this treatment is given over multiple fractions. This approach exposes minimal brain tissue to radiation, and does not preclude the use of WBRT if clinically warranted in the case of post-fSRS relapse. Using fractionated radiation, as opposed to a single fraction, takes advantage of the high α/β of lymphoma cells [12,13], and allows for treatment of a large tumour volume while sparing normal brain parenchyma.

fSRS presents a potentially promising treatment option for two distinct groups of patients with PCNSL for whom WBRT would usually be indicated. The first group is patients who refuse WBRT because of its anticipated side effects. The second group is older patients with a poor performance status, for whom the toxic effects of WBRT could be particularly morbid or life-threatening. This case series presents ten such cases to investigate whether providing targeted radiation may be a reasonable treatment option for patients with CNS lymphoma who decline WBRT or are too unwell to tolerate the toxicity of WBRT.

## 2. Materials and Methods

We retrospectively identified all patients treated with fSRS for CNS lymphoma from January 2010 to December 2020 at our institution. Patients who were not primarily treated with upfront chemotherapy or who did not complete the prescribed radiation treatment course were excluded. Descriptive statistics were used to summarize patient demographic data. Treatment details, the time from their last chemotherapy to completing fSRS, time from completion of fSRS to relapse or death, time from fSRS to WBRT, and other details, such as performance status and symptom burden, were collected from the patient chart and summarized. Recurrences were categorized as in-field or distant brain relapse after review of the follow-up MRI and a comparison of it to the original radiation treatment plan.

Treatment with fSRS was completed on a Cyberknife unit. The gross tumour volume (GTV) was outlined using enhancement on a T1-weighted MRI with a gadolinium contrast. The planning target volume was defined by expanding the GTV uniformly by 1 mm. The fSRS dose in all cases was 25 Gy in 5 fractions, prescribed to between the 67% and 82% isodose line, and delivered daily. Treatment was started within 7 days of MRI simulation. In cases where WBRT was given for relapse after fSRS, WBRT was delivered to a dose of 45 Gy in 25 fractions as previously described [14]. In cases where it was expected that this extended course of radiation could not be tolerated, a shorter course of 37.5 Gy in 15 fractions was prescribed. In cases of a very poor performance status, a dose of 20 Gy in 5 fractions was given.

In cases where identified patients had systemic lymphoma that relapsed in the CNS as the sole site of disease, our protocol was to follow the same treatment pattern as for PCNSL. Thus, such patients were included in this analysis, and comprise cases 3, 4, 6, and 10.

## 3. Results

A total of 10 patients were identified, who were treated with 14 courses of fSRS. The median age at the time of fSRS was 69 years (range: 56–88). After 14 fSRS treatments there were 10 distant brain recurrences among 6 patients, occurring on average 13.6 months after fSRS. A total of 4 of the 10 recurrences were treated with further fSRS, and 4 were treated with WBRT. There was one late in-field recurrence, which occurred after both fSRS and WBRT, and occurred 27 months after fSRS. The median survival after fSRS was 36 months. Individual cases are described as follows and summarized in Table 1.

### 3.1. Case 1

**Figure 1:**
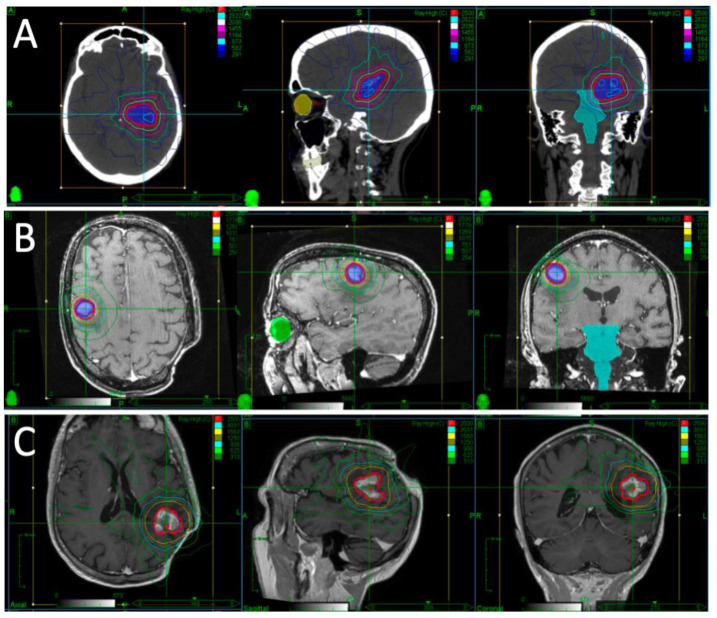
Target volumes and dose distribution for Case 1, outlining their first (**A**), second (**B**), and third (**C**) courses of fSRS. Repeat fSRS was necessary due to regional recurrences, but no in-field recurrences were noted in their case. The red isodose line represents the prescription dose of 25Gy.

A patient presented with falls, short-term memory loss and word-finding difficulties. An MRI revealed a 4.6 × 4.2 × 4.0 cm mass centered in the left thalamus. Due to the rapid progression of symptoms and development of hemiplegia, they underwent partial resection. Pathology revealed diffuse large B-cell lymphoma (DLBCL). They proceeded to receive two cycles of high-dose methotrexate and cytarabine but had progression while on chemotherapy, with MRI demonstrating a 1.8 × 1.2 cm enhancing lesion in the left middle cerebral peduncle and thalamus. Their KPS was 30–40. After multidisciplinary discussion, it was decided to proceed with fSRS to this lesion. Representative images of their fSRS are shown in Figure 1. fSRS was completed 3 months after their partial resection and 2 months after their last chemotherapy. They had a complete radiologic remission and regained much of their neurologic function, with some residual fatigue and right-sided weakness and sensory deficits.

Sixteen months after completion of their first fSRS, they developed left-sided weakness and an MRI revealed a mass in the superior right frontal lobe measuring 1.5 cm × 1.2 cm × 1 cm. After a discussion about WBRT or fSRS, they again opted to proceed with fSRS to this lesion, which was completed 17 months after their initial fSRS. They continued to have residual right-sided deficits that required a mobility aid and ongoing physiotherapy, but was otherwise stable.

Fifty-nine months after their second fSRS, they developed a seizure and imaging revealed a new enhancing lesion with moderate perifocal edema in the region of the surgical bed in the left posterior parieto-occipital region, measuring 2.0 × 1.8 × 2.2 cm. After discussion about WBRT or fSRS, they again opted to proceed with fSRS to this lesion that was completed 5 years after their second fSRS. One month later they developed vision loss and their optometrist noted extensive lymphoma in bilateral orbits that was then treated with radiation of a dose of 20 Gy in five fractions. They died 2 months after completing radiation to the orbits, and 79 months after their initial course of fSRS.

### 3.2. Case 2

A patient presented with confusion, memory loss and behavioral changes. Imaging revealed an enhancing parenchymal lesion involving the left side of the corpus callosum with periventricular enhancement on the right side, and an enhancing lesion within the left basal ganglia, suspicious for CNS lymphoma. A stereotactic biopsy revealed DLBCL. Staging was otherwise negative, though bone marrow biopsy did reveal a B-cell lymphoproliferative disorder in keeping with chronic lymphocytic leukemia. They completed four cycles of high-dose cyterabine and methotrexate. One month after chemotherapy, an MRI revealed a residual mildly enhancing area of T2 signal hypointensity centered in the left-sided corpus striatum and extending into the left cerebral peduncle. They were referred for radiation and, after discussion about WBRT, opted to proceed with fSRS to this region. They tolerated this with minimal side effects.

Five months after their first fSRS, imaging revealed a new 2.2 × 2.0 cm homogeneously enhancing mass in the right basal ganglia involving the caudate nucleus. They underwent a second course of fSRS to this lesion, completed 6 months after their first fSRS, and which they tolerated without any neurologic symptoms noted.

Nine months after their second fSRS, repeat imaging noted two new foci of contrast enhancement in the medial aspect of the left temporal lobe, measuring 2.9 mm and 3.8 mm. They again discussed WBRT and, being concerned about its long-term cognitive effects, proceeded to a third course of fSRS targeting both of these lesions, completed 10 months after the second course. They again tolerated this well with no neurologic side effects noted.

Six months after their third course of fSRS, while having minimal neurologic symptoms, imaging revealed significant disease progression with leptomeningeal enhancement in the left parietal lobe, right lentiform nucleus and left temporal lobe. They proceeded to have WBRT to a dose of 37.5 Gy in 15 fractions, completed 7 months after their third fSRS course.

Initially they developed short-term memory loss, confusion and expressive aphasia with no imaging changes. Their symptoms progressed to a decreased level of consciousness, complete aphasia, and severe L sided weakness, and imaging taken 10 months after WBRT revealed significant widespread disease progression, most notable in the bilateral cerebral convexities and posterior fossa structures. They died from progressive disease 1 year after WBRT.

### 3.3. Case 3

A patient presented with a mediastinal mass, biopsy-proven to be DLBCL. They received six cycles of CHOP-R (rituximab, cyclophosphamide, doxorubicin, vincristine, prednisone) then radiation to the involved site, 30.6 Gy in 17 fractions, due to residual disease. Two months after their mediastinal radiation they developed homonymous hemianopsia, and imaging revealed an enhancing mass in the left occipital lobe, abutting the left occipital bone and extending down to the level of the left tentorial leaflet, measuring 3.7 cm × 4 cm × 3.8 cm. It was unclear whether this represented a lymphoma or meningioma, and they underwent partial resection that confirmed DLBCL. They then underwent combination chemotherapy with four cycles of high-dose methotrexate and DHAP-R (dexamethasone, cytarabine, cisplatin, rituximab). The intent was to move forward with autologous stem-cell transplant, but they developed syncope and on imaging were found to have progressed on chemotherapy; an MRI revealed thick nodular enhancement along the surgical-margin posterior left occipital lobe, 3.2 × 1.4 × 1.7 cm, with a marked increase from the previous MRI.

They were referred for radiation and after discussing the possibility of WBRT, received fSRS to this region 1 month after their last chemotherapy. Their ECOG status at the time was 2. They tolerated their treatment well, but experienced mild chronic headaches. They continue to follow up with their hematologist 10 years after completing their fSRS with no clinical or radiologic signs of recurrence.

### 3.4. Case 4

A patient presented with a large retroperitoneal biopsy-proven DLBCL, with bone marrow involvement, for which they completed six cycles of CHOP-R chemotherapy. They had a complete response on their post-chemotherapy PET scan. Four months after completing chemotherapy, they developed weakness and vomiting, and a brain MRI revealed multiple parenchymal enhancing, diffusion-restricting lesions with the largest left cerebellar lesion resulting in mild to moderate supratentorial hydrocephalus. Extensive subependymal disease was present in both lateral ventricles, with contiguous involvement of the pituitary gland, infundibulum and hypothalamus. This was suspicious for DLBCL recurrence in the brain with leptomeningeal spread. They underwent MATRix (methotrexate, cytarabine, thiotepa, rituximab) chemotherapy followed by autologous stem-cell transplant. Their treatment was complicated by multiple DVTs, GI bleed, and ICU admission. A follow-up MRI found the almost complete resolution of their initial CNS disease, with two areas of residual enhancement involving the posterior third ventricle and cerebellum, alongside the development of a new 0.3 cm left insular juxtacortical focus of enhancement not seen on previous exams. Their ECOG status at that time was 4.

They were discharged from hospital and their functional status improved. The lesions, as viewed on on MRI, remained stable for 2 months, and they opted to proceed with fSRS. They were treated with 25 Gy in five fractions, completing radiation 4 months after their autologous stem-cell transplant. They remained healthy with no clinical or radiologic signs of disease recurrence 4 years after completion of fSRS, with no residual neurologic symptoms aside from mild intermittent headaches.

### 3.5. Case 5

A patient presented with confusion, fatigue, nausea, and expressive aphasia. Imaging revealed an irregular gyriform enhancement in the left frontal lobe measuring 5.9 × 4.9 × 3.8 cm, with dural abutment and thickening, in keeping with primary CNS lymphoma. They underwent resection, and pathology was consistent with DLBCL. Staging with CT of the chest, abdomen and pelvis and bone marrow biopsy revealed no extracranial disease. They were treated with two cycles of high-dose methotrexate followed by four cycles of methotrexate and cyterabine. An MRI taken after their last chemotherapy cycle demonstrated growth of their disease, revealing a lobulated homogeneously enhancing mass centered in the left frontal lobe cortical region, measuring 2.8 × 1.8 × 1.9 cm. They were referred for radiation and received fSRS 1 month after finishing their chemotherapy.

Fifteen months after their fSRS, the patient developed blurry vision in both eyes, and a vitreous biopsy was positive for lymphoma. An MRI did not reveal any progressive disease. They proceeded to WBRT of a dose of 45 Gy in 25 fractions, which was completed 16 months after fSRS. Acutely they developed severe conjunctival pain, and had significant short-term memory loss after WBRT. Four and a half years after WBRT they required transfer to an assisted living facility due to their cognitive decline. Ongoing MRI scans continued to show no recurrence of disease, but did demonstrate severe L frontal lobe atrophy. They died of pneumosepsis 6 years after completing WBRT, and 89 months after the completion of fSRS.

### 3.6. Case 6

A patient presented with a testicular mass. After orchiectomy, pathology was consistent with DLBCL, and no other sites of disease were noted on staging. They completed six cycles of CHOP-R, three cycles of high-dose methotrexate for CNS prophylaxis, and radiation to the contralateral testis of a dose of 30 Gy in 15 fractions. They were stable without any neurologic symptoms for several years. They then developed leg weakness and personality change, and a brain MRI taken 81 months after completion of chemotherapy revealed an irregular intra-axial enhancing mass in the left frontal lobe measuring 5.7 × 4.6 × 3.3 cm, and involving the anterior corpus collosum. They completed a course of steroids that resulted in a significant decrease in the size of the mass, consistent with a CNS relapse of their testicular lymphoma. Their KPS at the time was 90. They completed fSRS to this mass 82 months after completion of their chemotherapy.

Sixteen months after completion of their fSRS, they developed worsening confusion and their brain MRI revealed the recurrence of their disease outside of the treatment field diffusely throughout the posterior corpus collosum. Given the diffuse nature of their recurrence, they completed WBRT of a dose of 37.5 Gy in 15 fractions, 18 months after completing fSRS. There was notable cognitive decline after the completion of WBRT. Nine months after WBRT, there was increasing enhancement in their anterior corpus collosum, unknown whether to be from disease progression versus radiation-related changes; however, further growth on two later scans confirmed recurrence. This represented in-field recurrence 27 months after fSRS. They were started on palliative-intent ibrutinib, and died from disease progression 19 months after completing WBRT.

### 3.7. Case 7

A patient presented with weakness, dysphagia, anorexia, vision changes and panhypopituitarism. Imaging revealed a 2 cm suprasellar mass involving the hypothalamus and compressing the optic chiasm. They underwent a stereotactic biopsy, and pathology showed DLBCL. Further workup including bone marrow biopsy and CT staging were negative for extracranial disease. They started high-dose methotrexate monotherapy for six cycles. Eight months after finishing chemotherapy, imaging revealed an interval increase in the size of their infundibular lesion, measuring 0.5 × 0.6 × 0.7 cm. They were referred for radiation and completed fSRS 9 months after their last chemotherapy. They tolerated it well without any significant side effects. Five months after fSRS, they developed left-sided weakness, confusion and slurred speech. Reimaging revealed recurrence in the right corona radiata extending to the lateral basal ganglia. Their ECOG was 3–4. They were offered WBRT but declined any further therapy and instead transitioned to supportive care. They died 6 months after their fSRS.

### 3.8. Case 8

A patient presented with left-sided visual loss and imaging revealed gyriform thickening and enhancement in the right occipital lobe measuring 4.8 × 3.1 × 3.3 cm. They underwent resection and pathology revealed DLBCL. Staging did not reveal any other sites of involvement. They began high-dose methotrexate and completed five of a planned eight cycles, and stopped due to toxicities. MRI at that time revealed a significantly increased mass-like enhancement along the superior aspect of the resection cavity measuring up to 2.2 × 2.5 × 1.3 cm, consistent with progression, while on chemotherapy. They were referred for radiation and received fSRS to this region, 1 month after their last chemotherapy.

Four months after their fSRS they developed left-sided weakness and a decreased level of consciousness; their ECOG was 4, and MRI revealed the interval development of new confluent enhancement of corona radiata of the right parietal lobe, right ventricle, and right splenius of the corpus collosum, as well as hyperintensity of the right postcentral and precentral gyri. This was in keeping with widespread recurrence of their CNS lymphoma. After discussion about referral to palliative care, they did opt for WBRT to a dose of 20 Gy in five fractions that was completed 5 months after their fSRS, and they died 2 weeks after completing WBRT.

### 3.9. Case 9

A patient presented with left-sided weakness, ataxia and a fall and had a right cerebellopontine enhancing mass measuring 3.3 × 2.9 × 1.8 cm. They had a subtotal resection, with pathology revealing DLBCL. Systemic workup including CT chest/abdomen/pelvis and a bone marrow biopsy did not reveal any extracranial disease. They were treated with two cycles of high-dose methotrexate that was poorly tolerated due to mucositis. There was resolution of the residual tumour, but a new region of irregular enhancement at the superomedial aspect of the cavity measuring 1.5 × 0.4 × 1.3 cm, not seen in their immediate post-operative imaging, was concerning for progressive disease. Their KPS at time of consultation was 60.

They were treated with fSRS, completing their radiation 10 weeks after their subtotal resection and 3 weeks after their last chemotherapy. They tolerated their treatment well with minimal acute side effects. They continued to decline clinically with increasing confusion and fatigue, with brain MRI 1 month after fSRS showing no new or progressive disease or infarct. They were transferred to a hospice and died 2 months after fSRS.

### 3.10. Case 10

A patient presented with lower-limb cutaneous DLBCL. They were treated with five cycles of mini-CHOP-R chemotherapy, with good response. Fifteen months after chemotherapy they developed left sided weakness and imaging revealed a mass centered in the right periventricular white matter of the parietal lobe measuring 2.5 × 2.0 × 2.2 cm, as well as two additional lesions measuring 0.4 cm superior to their left lateral ventricle and 0.3 cm adjacent to their insular cortex. Systemic imaging was otherwise negative for extracranial disease. The lesions shrunk in response to steroids, with the smallest lesion disappearing entirely, in keeping with recurrence of their DLBCL. They were referred to radiation oncology with the purpose of providing symptom palliation. They received fSRS to the two larger lesions. They continued to decline after treatment, were transferred to a hospice, and died 2 months after their fSRS.

## 4. Discussion

Here, we presented ten cases of CNS lymphoma treated with fSRS. While the specifics of each case presentation vary, they are unified by the desire on behalf of the patient and treating physician to avoid WBRT, while still having the option to use WBRT as a salvage therapy. Rates of distant brain relapse after fSRS were high, occurring after 10 of the 14 courses of fSRS. Cases 1 and 2 demonstrate that while distant brain relapse can be effectively treated with fSRS, further recurrence is still likely.

Four of the ten cases did proceed to WBRT after distant brain relapse, and significant side effects were noted in every case. Cases 2, 5 and 6 demonstrate that WBRT can be delayed for over a year with the use of one or several courses of fSRS that are generally much better tolerated than WBRT. In addition, cases 3 and 4 demonstrate that WBRT is not necessary in all cases of residual CNS lymphoma, as these patients have lived without progression for 4 and 10 years after fSRS.

Prior study of WBRT as a consolidative treatment for PCNSL had noted extensive neurocognitive toxicity in long-term follow-up. In RTOG 9310, 10% of patients died as a result of long-term neurotoxicity in this regimen, which was the most common cause of death among the long-term survivors of the trial [4,14]. Since then, there has been extensive interest in mitigating this toxicity. When WBRT was omitted as part of the G-PCNSL-SG-1 study, patients who avoided WBRT had approximately half the rate of clinically apparent neurotoxicity (49% vs, 26%), but omitting WBRT resulted in significantly worse progression-free survival [5,15]. Recent trials such as PRECIS and IESLG2 have investigated the use of ASCT to replace WBRT as consolidative treatment for CNS lymphoma. WBRT in this context resulted in worse attention and executive function [16], as well as worse balance and neurocognitive deterioration [8,17]. While there has also been prospective research investigating a decreased dose of WBRT to mitigate these side effects [6,7], there has been no prospective trial of partial-brain irradiation in the context of PCNSL.

Older retrospective analysis of partial-brain irradiation for CNS lymphoma demonstrated high rates of both in-field recurrence and distant brain relapse, of 57% and 49%, respectively [18]. While our rates of distant brain relapse are comparable to those previously reported, we observed only one case of in-field recurrence, occurring 27 months after fSRS. This difference likely reflects the improvements in imaging and radiation-targeting technology that allow for the improved delineation of target lesions. Our case series is limited by its sample size and the potential for selection bias of patients who were offered and completed therapy. However, these improvements compared to previous trials may indicate that fSRS, delivered using modern treatment techniques, should be investigated in a prospective manner to determine its place in the treatment of CNS lymphoma.

CNS lymphoma has a poor prognosis, and treatment should balance the goals of achieving local control while maintaining quality of life. This case series presented two patient populations for which fSRS was given for CNS lymphoma. For healthier patients or those with minimal residual disease, such as cases 1 through 6, fSRS was used to delay or avoid WBRT; in some cases, fSRS achieved the prolonged remission or cure for residual disease after chemotherapy. For morbid patients with a more guarded prognosis, such as those in cases 7 through 10, fSRS was used to avoid the cognitive side effects of WBRT as these patients approach end-of-life from progressive or comorbid disease. Based on this experience, we feel that fSRS may be a reasonable treatment option to explore for select patients with CNS lymphoma wishing to avoid WBRT.

## 5. Conclusions

The treatment of CNS lymphoma with fSRS resulted in high rates of local control and avoided or delayed the toxic effect of WBRT. However, rates of distant brain relapse were high. fSRS may be a reasonable treatment option for future study in patients with CNS lymphoma who decline WBRT or for whom the toxic effects of WBRT would be poorly tolerated.

## Figures and Tables

**Table 1:** Summary of cases.

Case #	Resection	Initial Chemotherapy	Time from Last Chemo to 1st fSRS (mo)	Time to Distant Brain Relapse (mo)	Salvage Treatment	Time to Local Recurrence (mo)	Time from 1st fSRS to Death (mo)	Time from fSRS to Last Follow-Up, If Living (mo)
1	Yes	HD methotrexate and cyterabine 2 cycles	2	16	fSRS	N/A	79	N/A
				59	fSRS	N/A		
				1	Orbit RT	N/A		
2	No	HD methotrexate and cyterabine 4 cycles	2	5	fSRS	N/A	36	N/A
				9	fSRS	N/A		
				6	WBRT	N/A		
3	Yes	CHOP-R 6 cycles, then DHAP-R and HD methotrexate 4 cycles	1	N/A	N/A	N/A	N/A	120
4	No	CHOP-R 6 cycles, then MATRix	4	N/A	N/A	N/A	N/A	48
5	Yes	HD methotrexate 2 cycles, then methotrexate and cyterabine 4 cycles	1	15	WBRT	N/A	89	N/A
6	No	CHOP-R 6 cycles, HD methotrexate 3 cycles	82	16	WBRT	27	36	N/A
7	No	HD methotrexate 6 cycles	9	5	BSC	N/A	5	N/A
8	Yes	HD methotrexate 5 cycles	1	4	WBRT	N/A	5	N/A
9	Yes	HD methotrexate 2 cycles	1	N/A	N/A	N/A	2	N/A
10	No	R-mini-CHOP 5 cycles	16	N/A	N/A	N/A	2	N/A

HD = high-dose; CHOP-R = rituximab, cyclophosphamide, doxorubicin, vincristine, prednisone; DHAP-R = dexamethasone, cytarabine, cisplatin, rituximab; MATRix = methotrexate, cytarabine, thiotepa, rituximab; fSRS = fractionated stereotactic radiosurgery; WBRT = whole-brain radiotherapy; RT = radiotherapy; BSC = best supportive care.

## Data Availability

The data presented in this study are available in our results, and no additional data sharing is applicable to this study.
